# Supplementary material for: Sub-second ultrafast yet programmable wet-chemical synthesis
Source: Nat Commun. 2023 Aug 18;14:5015. doi: 10.1038/s41467-023-40737-5 (PMC10439120; doi:10.1038/s41467-023-40737-5)
Supplement: Supplementary file 1 — Supplementary Information [file 41467_2023_40737_MOESM1_ESM.pdf]

Supplementary Information

Sub-second ultrafast yet programmable wet-chemical synthesis

Zhang et al.

Contents

Supplementary Fig. 1 Stability of the GF ··· 4
Supplementary Fig. 2 Contact angles of GF towards common solvents ··· 5
Supplementary Fig. 3 Optical images of the cross-section of GF before and after the addition of the solvent ··· 6
Supplementary Fig. 4 Temperature evolution of the cooling process after cutting off the current supply ··· 7
Supplementary Fig. 5 Simulation of the WIJH process ··· 8
Supplementary Fig. 6 Average temperature evolution and the normalized height of the solution during the simulated bulk heating process ··· 10
Supplementary Fig. 7 Schematic of the WIJH setup ··· 11
Supplementary Fig. 8 The separation of HKUST-1 film from the GF using a tape ··· 12
Supplementary Fig. 9 XRD patterns of HKUST-1/GF prepared within different duration times ··· 13
Supplementary Fig. 10 ICP-MS test for the quantification of the copper element ··· 14
Supplementary Fig. 11 N_2 adsorption/desorption isotherms ··· 15
Supplementary Fig. 12 SEM images of HKUST-1/GF prepared by WIJH within different times ··· 16
Supplementary Fig. 13 SEM images of HKUST-1/GF samples obtained by bulk Joule heating under different temperatures ··· 17
Supplementary Fig. 14 The relationship between WIJH nucleation rate and initial concentrations of precursors ··· 18
Supplementary Fig. 15 The relationship between WIJH growth rate and growth temperature ··· 19
Supplementary Fig. 16 SEM images of HKUST-1/GF prepared at room temperature with different times ··· 22
Supplementary Fig. 17 SEM images of HKUST-1/GF obtained by solvothermal method with different times ··· 23
Supplementary Fig. 18 SEM images of HKUST-1/GF obtained by bulk Joule heating with different times ··· 24
Supplementary Fig. 19 SEM images of HKUST-1/GF obtained by the evaporation at room temperature with different times ··· 25
Supplementary Fig. 20 SEM images of products obtained by oven evaporation with different times ··· 26
Supplementary Fig. 21 XRD patterns of the product obtained by oven evaporation ··· 27
Supplementary Fig. 22 SEM images of HKUST-1/GF obtained with different pulse cycles ··· 28

Supplementary Fig. 23 SEM image of the product obtained by the solvothermal method within ultralow precursor concentrations ··· 29
Supplementary Fig. 24 SEM images of HKUST-1/GF obtained within different initial concentrations of the precursors ··· 30
Supplementary Fig. 25 XRD patterns of HKUST-1/GF prepared within different initial concentrations of the precursors ··· 31
Supplementary Fig. 26 SEM images of HKUST-1 nanoparticles and microparticles obtained by WIJH ··· 32
Supplementary Fig. 27 WIJH synthesis and characterizations of MIL-88A(Fe)/GF ··· 33
Supplementary Fig. 28 WIJH synthesis and characterizations of Tb-BTC/GF ··· 34
Supplementary Fig. 29 FT-IR spectra of TAPB-DMTA/GF and GF ··· 35
Supplementary Fig. 30 WIJH-based continuous fabrication ··· 36
Supplementary Fig. 31 WIJH-based portable fabrication ··· 37
Supplementary Fig. 32 HKUST-1/GF synthesized by the solvothermal method for IJH-controlled capture and liberation of CO_2 ··· 38
Supplementary Fig. 33 Schematic diagram of the custom-designed IJH setup for the CO_2 adsorption/desorption analysis ··· 39
Supplementary Fig. 34 TGA curves of the precursors and product for HKUST-1 ··· 40
Supplementary Table 1 Analysis results of the N_2 adsorption/desorption isotherms ··· 41
Supplementary Table 2 Comparison of WIJH strategy for the synthesis of MOF films with other synthesis methods reported in the literature ··· 42
Supplementary Table 3 Statistic results of the synthesis of HKUST-1 on the GF via different heating strategies ··· 44
Supplementary References ··· 45

1. Supplementary Figures and Discussion

Supplementary Discussion 1

GF is stable at 600 °C in air without obvious chemical damage and thus weight loss. No structure changes were found in Raman spectra before and after the Joule heating (Supplementary Fig. 1).

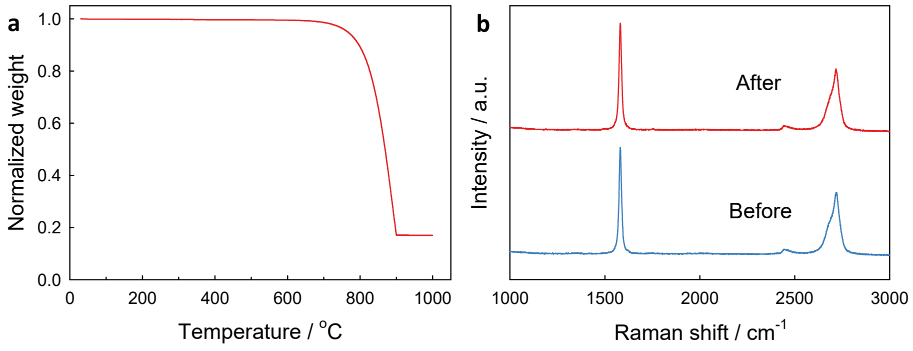

Supplementary Fig. 1 Stability of the GF. a) TGA curve in air, b) Raman spectra before and after the Joule heating of a pulse of 3A for 0.95 s.

Supplementary Discussion 2

The good wettability of GF towards common solvents enables the formation of the thin liquid film, as proved by small contact angles in the range of 25.6 - 36.8º (Supplementary Fig. 2). Compared with bulk solution, such a thin layer would not only accept the heat sufficiently and rapidly via a large heating area, but also spatially confine the heat around GF, leading to a significant drop in thermal losses by the bulk and a boost in the thermal efficiency.

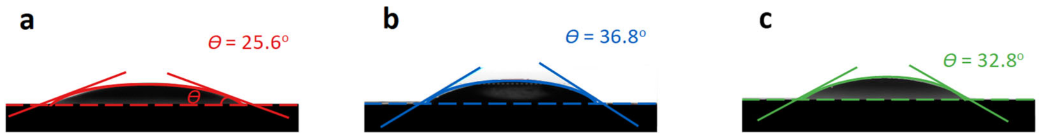

Supplementary Fig. 2 Contact angles of GF towards common solvents for bottom-up wet-chemical synthesis. a) The mixture of H_2O, EtOH, and DMF (1:1:1) on GF. b) H_2O on GF treated by air plasma. c) The mixture of H_2O, MeOH, and DMF (1:1:2) on GF.

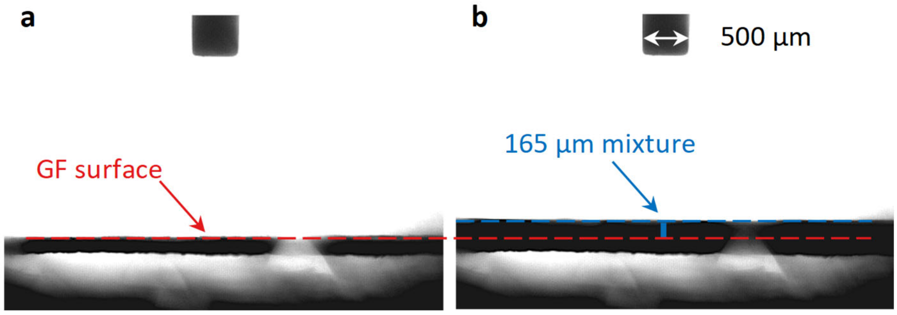

Supplementary Fig. 3 Optical images of the cross-section of GF a) before and b) after the addition of 2 μL of the mixture of H_2O, EtOH, and DMF (1:1:1). The height of the liquid film was measured by comparing the photographs before and after the spreading of the liquid on the GF.

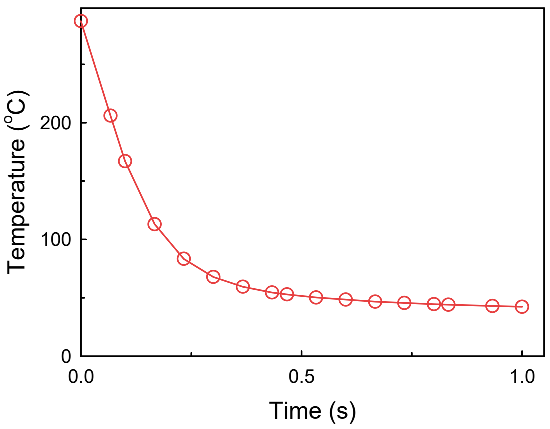

Supplementary Fig. 4 Temperature evolution of the cooling process after cutting off the current supply of a d.c. pulse of 3 A for 0.95 s.

Supplementary Discussion 3

Through the direct and fast heat transfer from the GF to the liquid film layer (Supplementary Fig. 5-a), the solution was instantaneously heated with a WIJH process (Supplementary Fig. 5-b), and the heat is confined around the GF (Supplementary Fig. 5-c). Evaporation was clearly observed, as proved by the sharply reduced height of the liquid film with increased time.

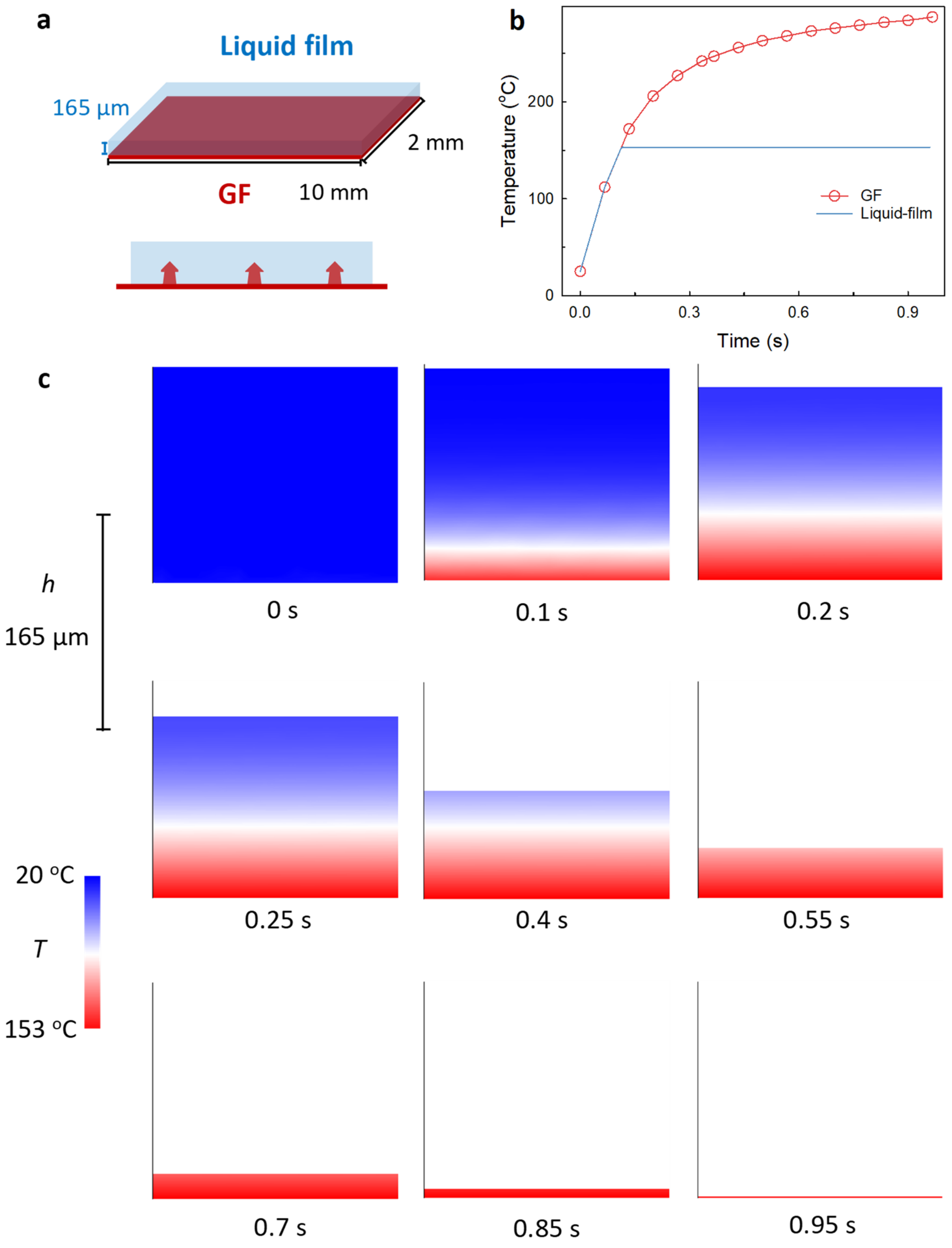

Supplementary Fig. 5 Simulation of the WIJH process (a pulse of 0.95s, 3 A). a) Schematic of the system and its cross-section that indicates the direction of heat transfer. b) Temperature evolution of GF recorded by a high-speed infrared thermometer, and the maximum temperature evolution of the liquid film layer. c) Thermal distribution of

the liquid-film layer when t equals 0, 0.1, 0.2, 0.25, 0.4, 0.55, 0.7, 0.85, and 0.95 s. Source data are provided in the Source Data file.

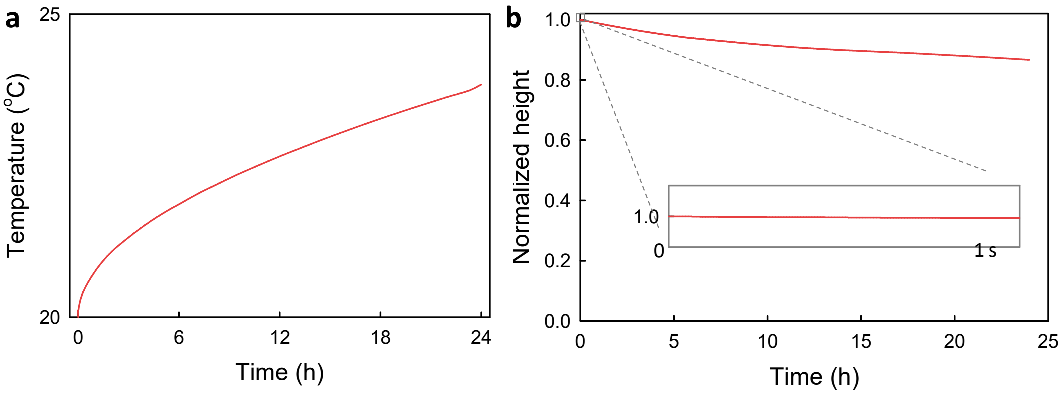

Supplementary Fig. 6 a) Average temperature evolution and b) the normalized height of the solution during the simulated bulk heating process.

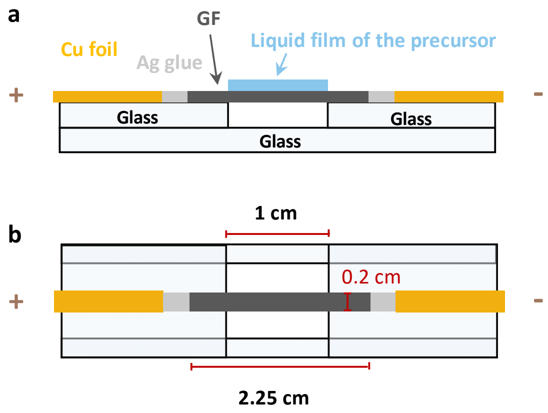

Supplementary Fig. 7 a) Side view and b) top view of the setup schematic for the WIJH synthesis.

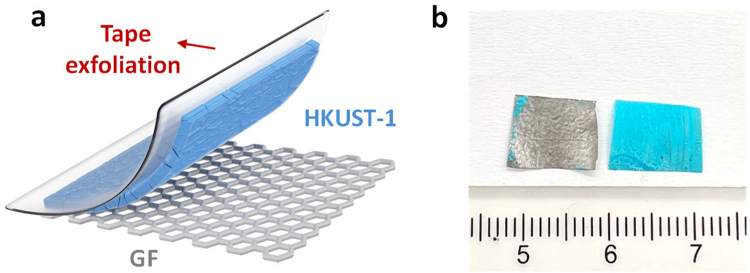

Supplementary Fig. 8 a) Schematic of the separation of the product of HKUST-1 film from the GF substrate using a tape, and b) photographs of the separated GF (left) and HKUST-1 film (right).

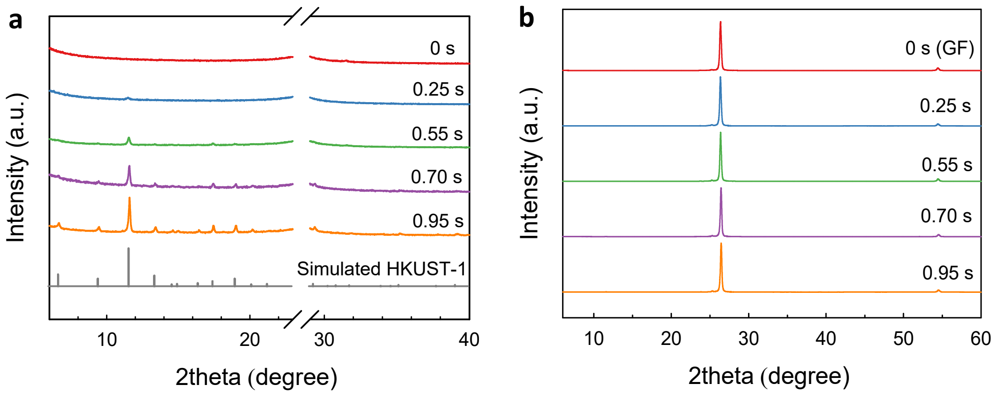

Supplementary Fig. 9 XRD patterns of simulated HKUST-1 and HKUST-1/GF prepared within different duration times in the 2θ range of a) 5-40° and b) 5-60°, displaying different characteristic peaks of HKUST-1 and graphene. Two diffraction peaks around 26.4°and 54.5° are indexed to graphene.

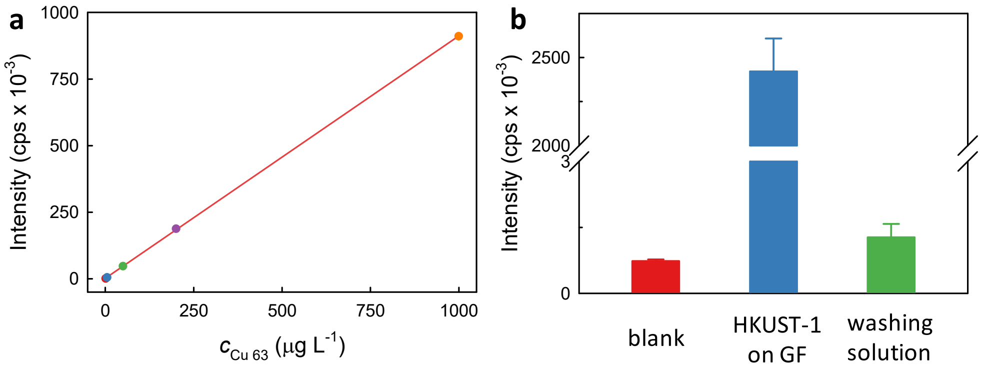

Supplementary Fig. 10 ICP-MS test for the quantification of the copper element. a) Calibration curve of the standard samples. b) Quantification results of different samples. The concentrations of copper in these samples were (0.26 ± 0.04), (2348.60 ± 188.43), and (0.72 ± 0.33) μg L^{-1}, respectively. Error bars represent the standard deviations of measurement from three samples.

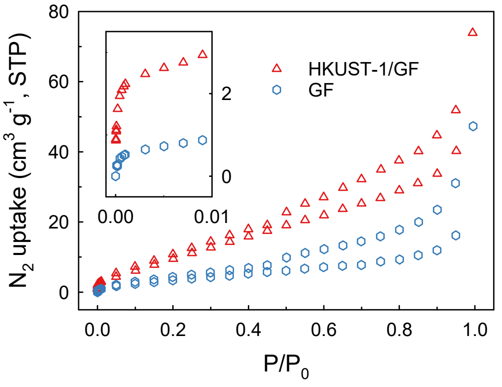

Supplementary Fig. 11 N_2 adsorption/desorption isotherms of HKUST-1/GF and GF.

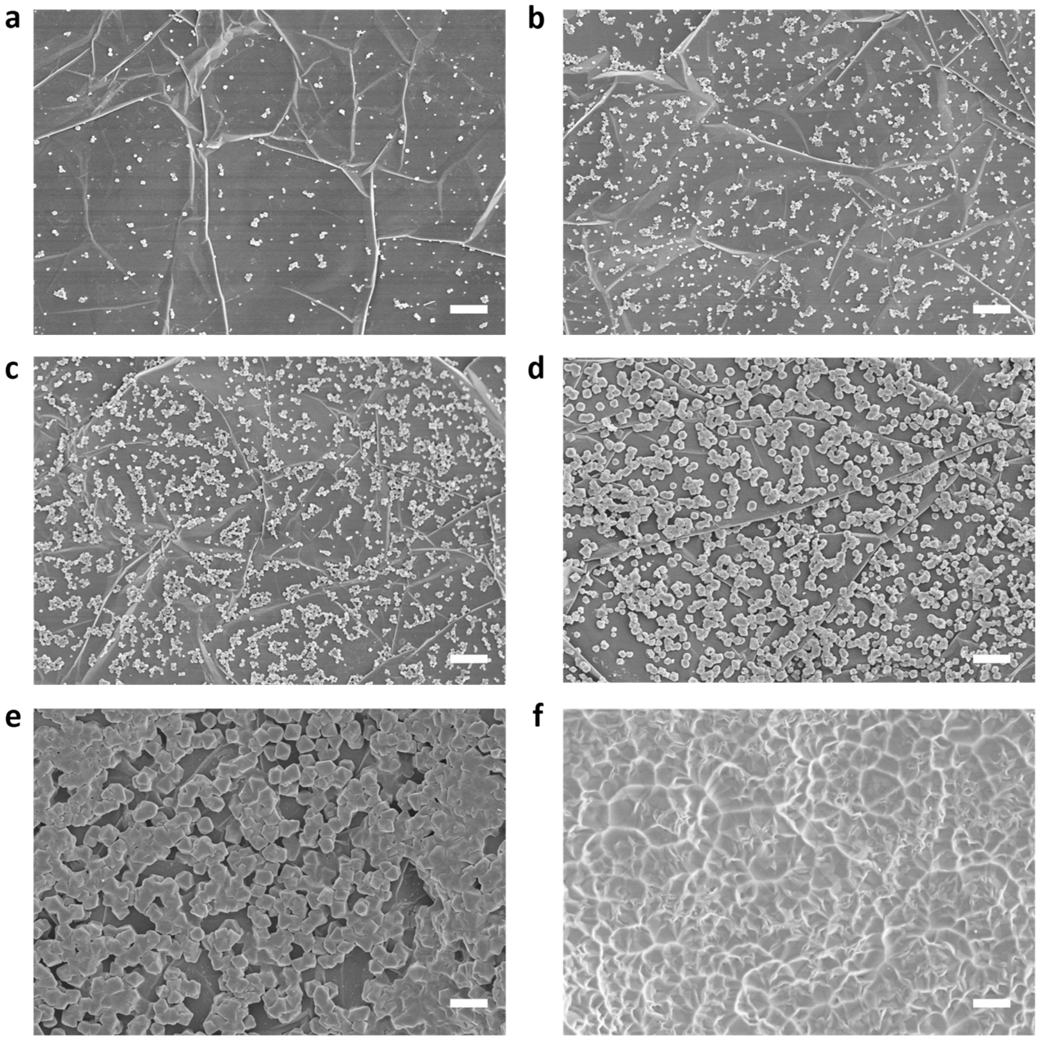

Supplementary Fig. 12 SEM images of HKUST-1/GF obtained by WIJH with a) 0.25 s, b) 0.4 s, c) 0.55 s, d) 0.7 s, e) 0.85 s, f) 0.95 s. Scale bar: 2 μm.

Supplementary Discussion 4

Heating (temperature effect) and evaporation (concentration effect) are the two most essential factors in the confined interfacial heating mechanism for ultrafast yet programmable synthesis. They affect and are coupled together along the WIJH process, synergistically accelerating and controlling the nucleation and growth on the GF. Specifically, they display different characteristics in different stages: a rapidly increased temperature caused by Joule heating in incubation-I, an evaporation-caused reduction of the solvent and the corresponding sharply elevated monomer concentration in nucleation-II, and another dramatically increased temperature in growth-III. To confirm their effects in the crystallization, a series of control experiments were conducted, using the typical WIJH synthesis of HKUST-1/GF under 3 A as the model.

Incubation stage I (the initial 0.25 s): Joule-heating-caused high temperature around the GF was expected to promote the crystallization, particularly in the initial stage with limited evaporation. To confirm its effect, the bulk Joule heating under different temperatures were conducted by applying different current intensities to the immersed GF in the bulk solution (85 mM $Cu(NO_3)_2$ and 55 mM H_3BTC). In that case, the evaporation-caused concentration could be negligible due to the large-volume solution of 200 μL and the short heating time of 5 s. As shown in Supplementary Fig. 13, as the final temperature of the GF increased from 240 to 450 °C, more and bigger particles appeared on the GF. This demonstrated the Joule-heating-induced crystallization and highlighted one of the advantages of interfacial heating to control the reaction around the GF.

Nucleation stage II (0.25-0.55 s): According to the following classic nucleation equation, the largest effect on nucleation rate comes from supersaturation (related to concentration)[1]. In our case, the intense evaporation in the nucleation stage leads to a sharp decrease of the normalized height of the liquid film to 0.21 (Fig. 2b), which was expected to elevate the supersaturation dramatically, and thus cause the nucleation burst. To simulate the evaporation-caused concentration effect, the nucleation rates within different initial concentrations of the precursors were recorded and compared. As shown in Supplementary Fig. 14, the average nucleation rates increased from 85.5 to 318.2 nm s^{-1}, as the concentrations of $Cu(NO_3)_2$ increased from1.7 to 85 mM (all with the same 3:2 molar ratio relative to H_3BTC). It confirms the concentration effect in the nucleation stage.

Growth stage III (0.55-0.95 s): As the rapidly-increased temperature is the most remarkable factor in this stage, the temperature effect was investigated by collecting the products within the same nucleation conditions but at different growth temperatures (Supplementary Fig. 15). The experiments were conducted by programming the electrified procedures of an initial pulse of 3 A for 0.55 s for nucleation, following by different current intensities to achieve different temperatures (Supplementary Fig. 15-

a). The average growth rates increased from 227.5 to 1697.5 nm s^{-1}, as the final growth temperature increased from 166 to 289 ºC. It confirms that temperature plays a critical role in the growth.

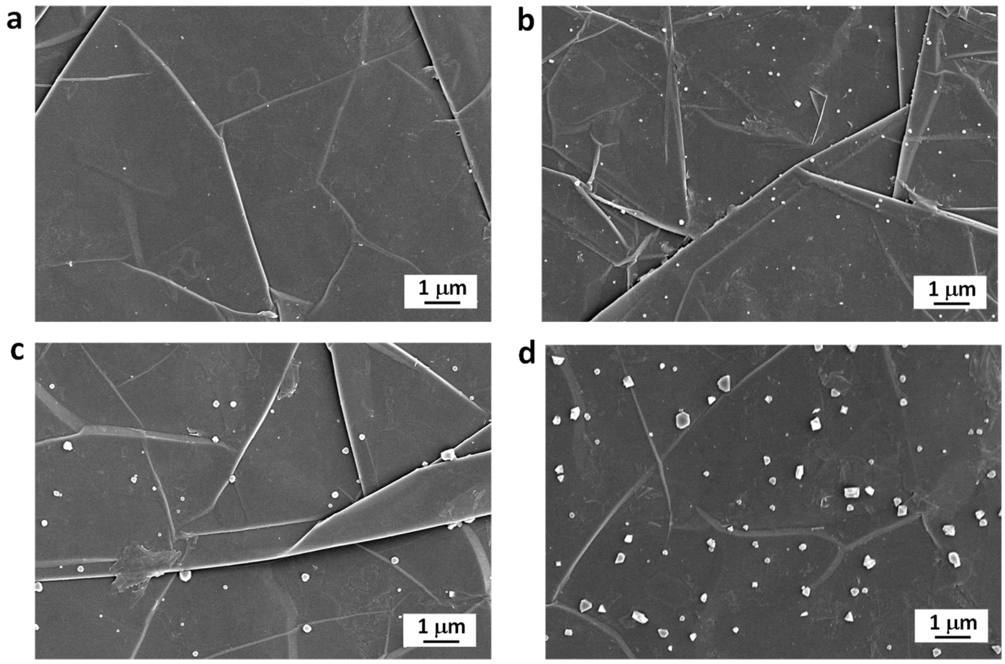

Supplementary Fig. 13 SEM images of HKUST-1/GF samples obtained by bulk Joule heating under different temperatures of a) 240 ºC, b) 300 ºC, c) 380 ºC, and d) 450 ºC.

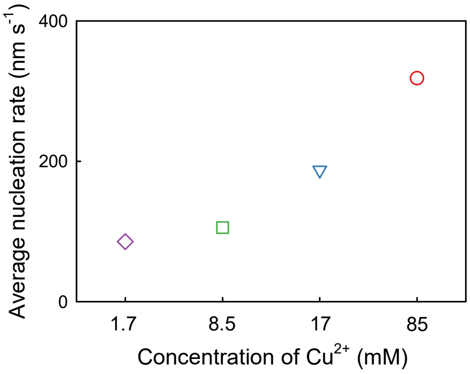

Supplementary Fig. 14 The relationship between nucleation rate and initial concentrations of precursors (with the same 3:2 molar ratio of $Cu(NO_3)_2$ and H_3BTC).

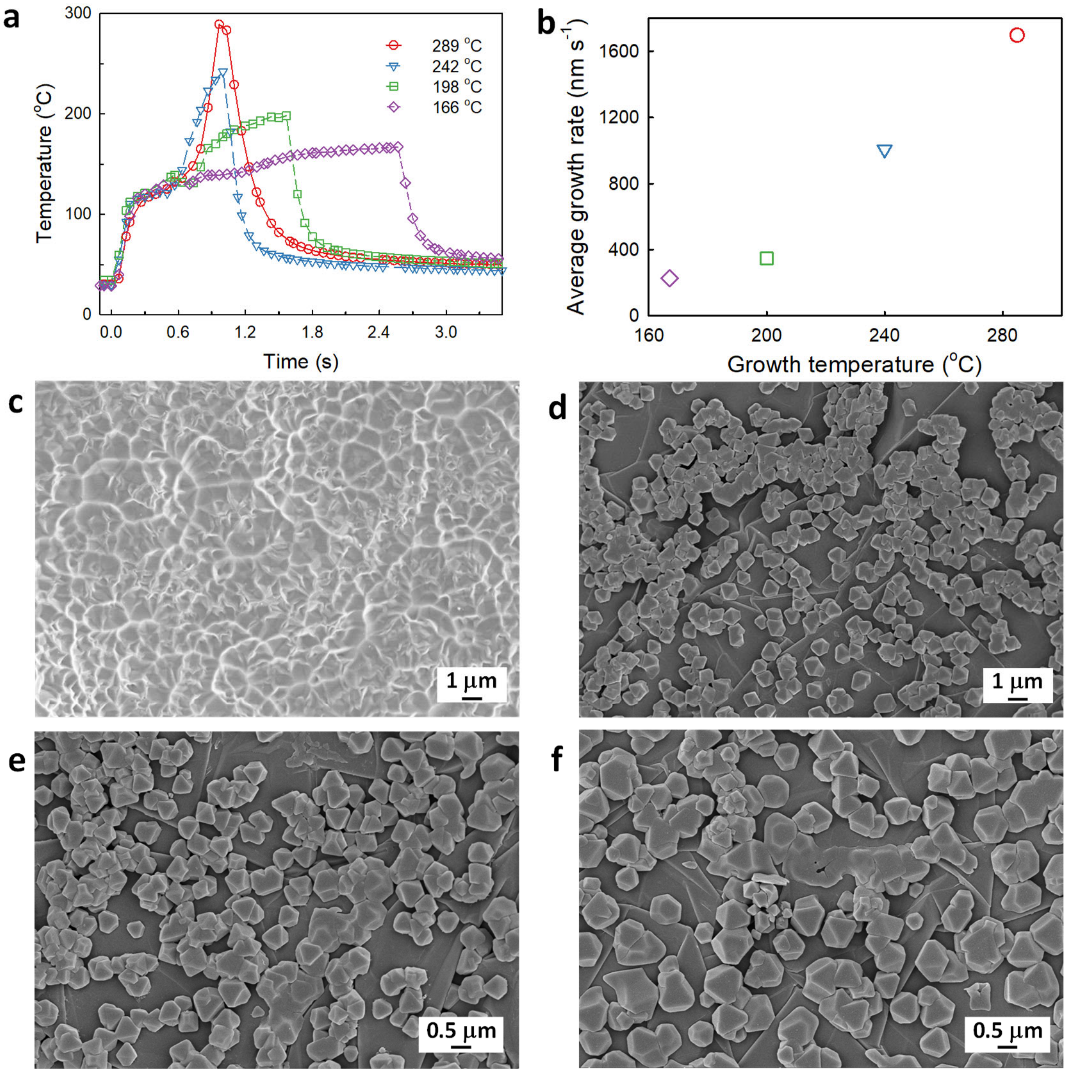

Supplementary Fig. 15 a) Temperature profiles of the WIJH synthesis within different growth temperatures. b) The relationship between growth rate and growth temperature. SEM images of the corresponding products of HKUST-1/GF obtained with different growth temperatures of c) 289 ºC, d) 242 ºC, e) 198 ºC and d) 166 ºC.

Supplementary Discussion 5

A series of control experiments were systematically performed to verify and compare that the confined interfacial heating is the driving force towards ultrafast synthesis. Reaction conditions include:

C1) blank: GF was immersed in 400 μL precursor solution, and then sealed and incubated at room temperature without evaporation of the solvent.

C2) solvothermal synthesis: GF was immersed in 400 μL precursor solution, and then sealed and incubated in an oven at 120 °C.

C3) bulk Joule heating: GF was immersed in 400 μL precursor solution, and then subjected to a Joule heating process (3 A).

C4) WIJH: GF was coated by 2 μL precursor solution, and then subjected to a Joule heating process (3 A).

C5) evaporation: GF was coated by 2 μL precursor solution, and then incubated in the open environment at room temperature or in a heating oven at 120 °C.

Compared with a few small particles (115-185 nm) obtained at room temperature with a long reaction time (Supplementary Fig. 16), the crystallization is clearly accelerated under heating, as proved by the remarkedly-increased particle density and size at C2)-C4) (Figs. 1f and 2d, and Supplementary Figs. 17-18, 20). The detailed comparisons of C2)-C4) in Table S3 indicate the remarkedly enhanced efficiency via confined interfacial heating. C2) represents the most widely adopted heating strategy in wet-chemical synthesis. As the bulk solution serves as the heat source or heat transfer intermedium, the thermal field of the reaction system is determined by its heat-relevant properties. The bulk nature and low boiling point limit the ramping rate and the reaction temperature. At the same time, conventional heaters with poor heat transfer and large thermal inertia further worsen the heating efficiency. Moreover, with an inert surface with limited nucleation sites, the homogeneous nucleation in bulk is prior during the near-equilibrium heating, as proved by more blue precipitations in the solution (Inset of Supplementary Fig. S17-f). This competitively consumed the precursors, further hindering the heterogeneous nucleation on the GF. Besides, the crystallization of MOFs always follows a slow curve in LaMer model with simultaneous nucleation and growth, leading to a large variability in crystal size. In the final cooling stage, the bulk solution presents a low cooling rate, which further increases the time and reduces the controllability towards the reaction.

The above disadvantages were partially overcome by powerful Joule heating technology. In C3), benefiting from low heat capacity and high interfacial heat transfer rate, the GF presents remarkable ramping and cooling rates. As the Joule heat was generated and transferred from the GF to the solution, the high temperature around the GF could help overcome the energy barrier to induce heterogeneous nucleation. Besides, more distorted octahedrons, rather than regular octahedron crystals (Supplementary

Figs. 17-e and 17-f), were found in Supplementary Figs. 18-c and 18-d, which indicates the heat-induced growth on the confined plane. Nonetheless, the efficiency is far from that of the WIJH, highlighting the critical effect of the thin layer for rapid ramping and evaporation.

In C5), with a trace solution of 2 μL for evaporation at room temperature, more small particles around 25 nm at 12 h (Supplementary Figs. 19-e and 19-f)) were found when compared with that of C1). Under heating at 120 °C, the evaporation rate is significantly promoted, but a dried and uneven blue film with unclear microstructure was obtained at 10 min (Supplementary Figs. 20-e and 20-f)). In addition to the characteristic peaks indexed to HKUST-1, a new peak at 12.8° that can be assigned to the (001) plane of copper hydroxide nitrate was found (Supplementary Fig. 21). This could be the heating product of copper nitrate[2], indicating a precipitation event during this evaporation condition. Ideally, the evaporation accelerates the crystallization with the concentrated precursors. However, it requires a rate match between the crystallization and the evaporation-caused precipitation. In WIJH, the high crystallization rate ensures the rapid consumption of the precursors, avoiding the precipitation of the precursors and thereby obtaining intact products.

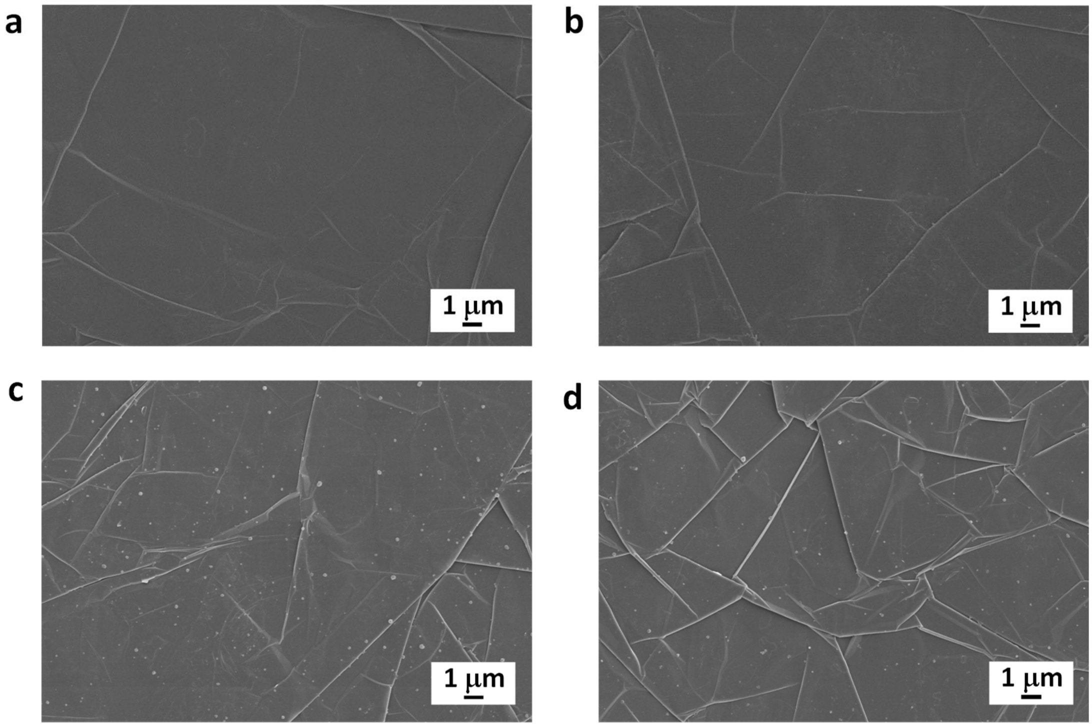

Supplementary Fig. 16 SEM images of HKUST-1/GF prepared at room temperature with a) 1 min, b) 10 min, c) 60 min, and d) 12 h.

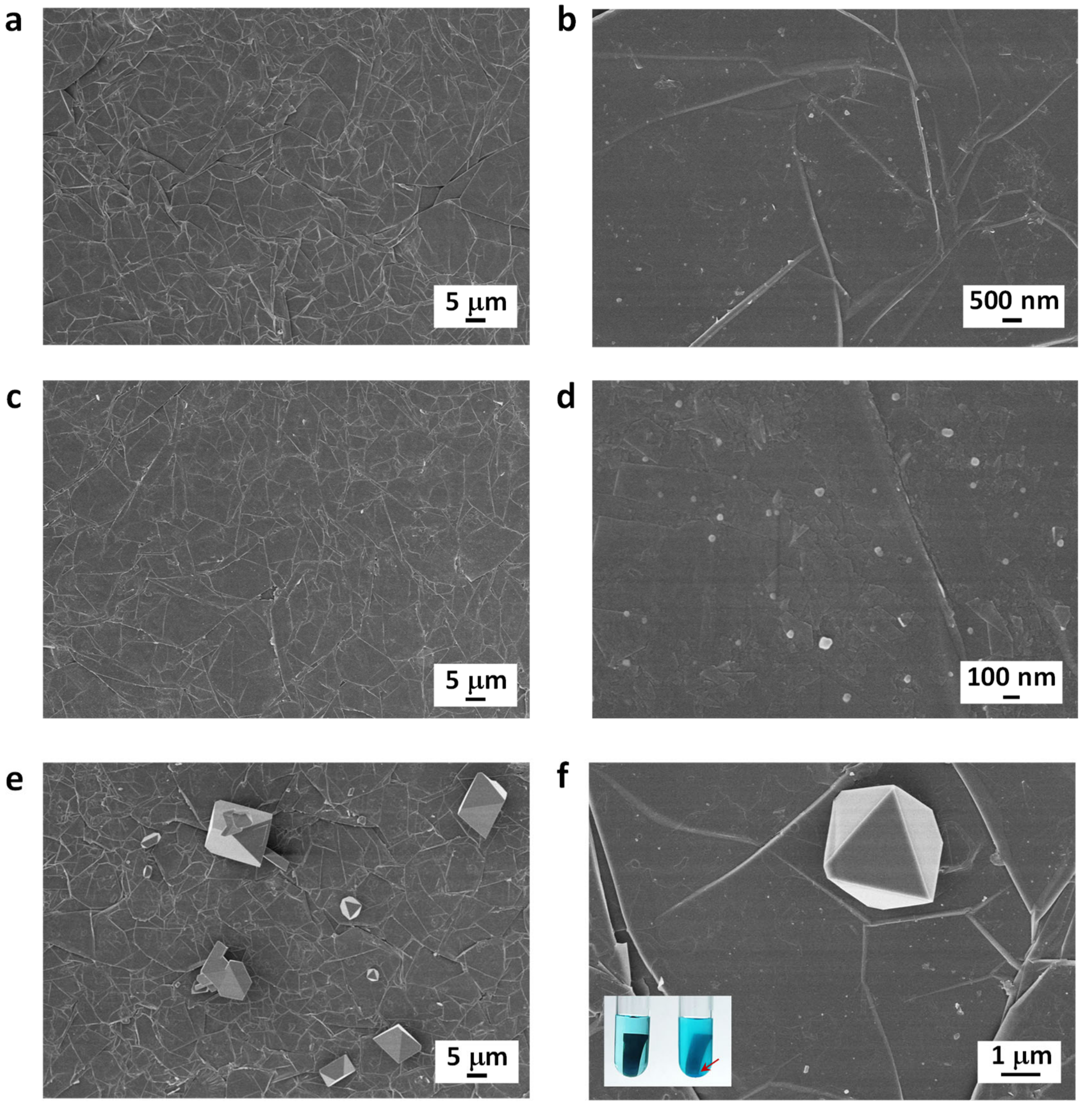

Supplementary Fig. 17 SEM images of HKUST-1/GF obtained by solvothermal method with a-b) 1 min, c-d) 10 min, e-f) 60 min. The inset of f is the photographs of the solution before (left) and after (right) the synthesis, and the red arrow indicates the spontaneous blue precipitation of large HKUST-1 particles.

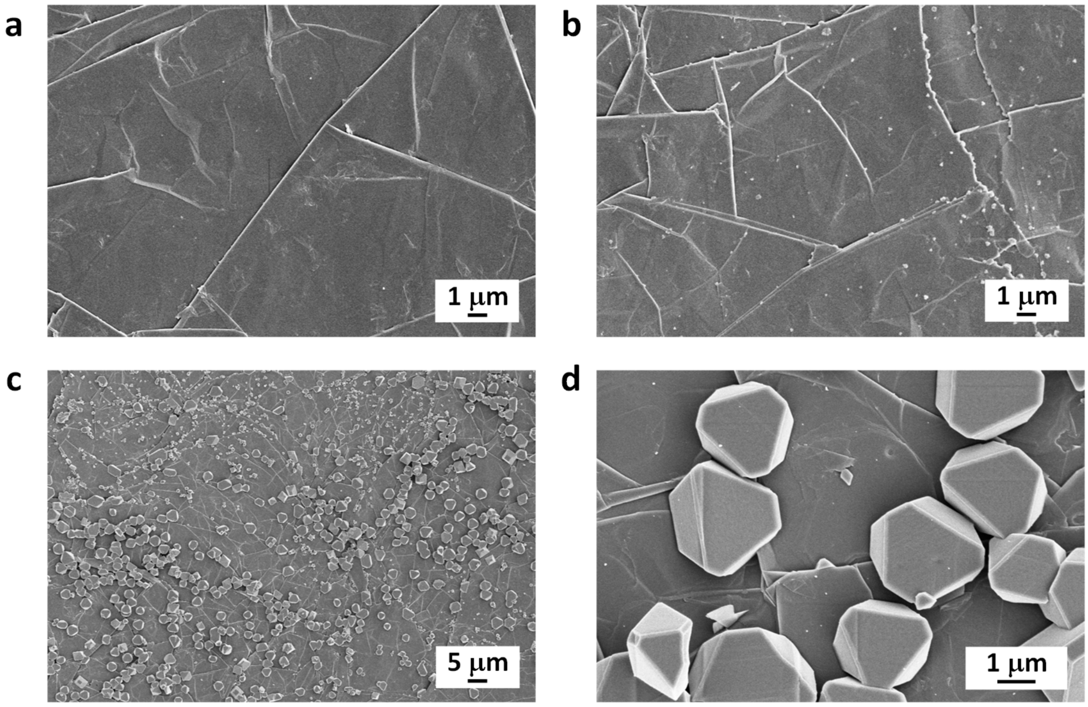

Supplementary Fig. 18 SEM images of HKUST-1/GF obtained by bulk Joule heating with a) 0.95 s, b) 30 s, and c-d) 2 min.

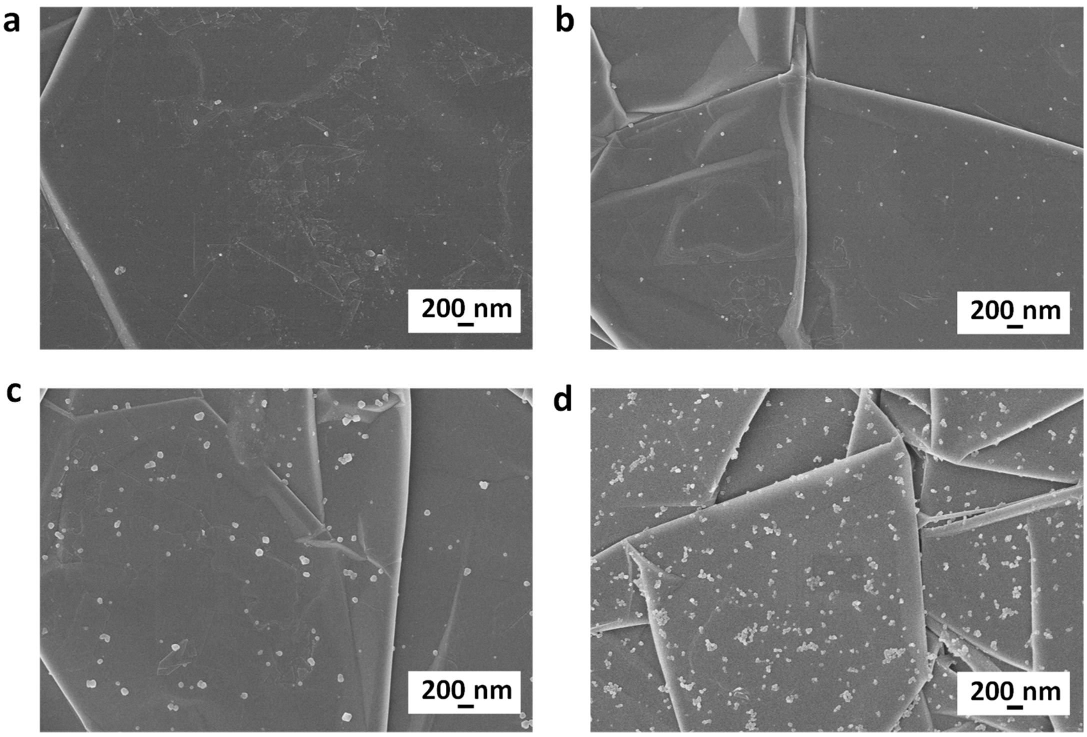

Supplementary Fig. 19 SEM images of HKUST-1/GF obtained by the evaporation at room temperature for a) 1 min, b) 10 min, c) 60 min, and d) 12 h.

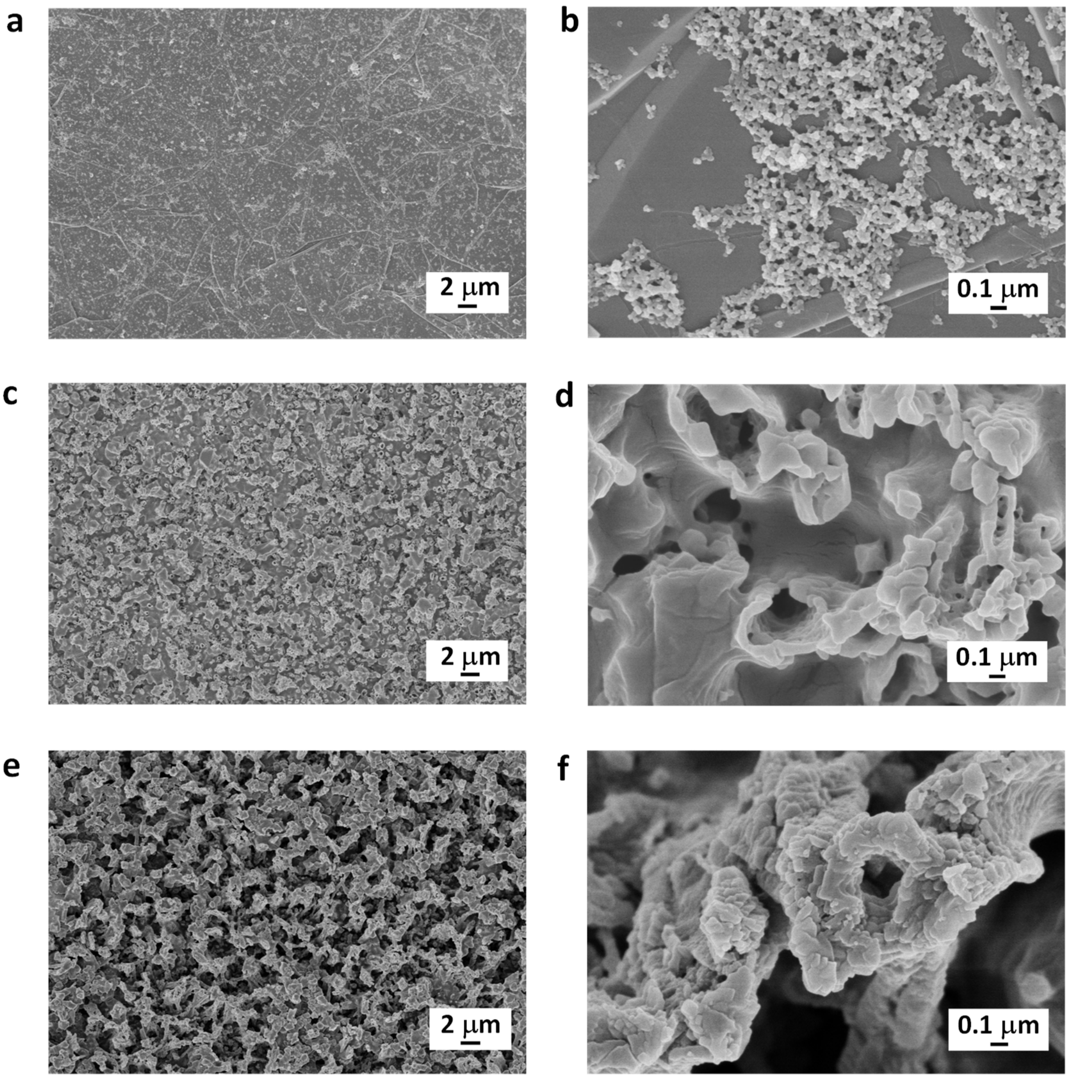

Supplementary Fig. 20 SEM images of products obtained by evaporation in an oven at 120 ºC with a-b) 1 min, c-d) 10 min, and e-f) 60 min.

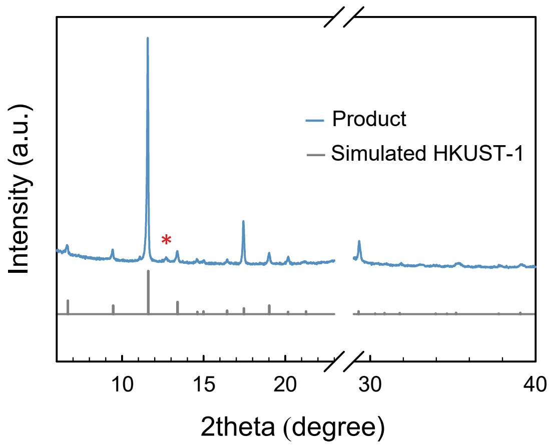

Supplementary Fig. 21 XRD patterns of simulated HKUST-1 and the product obtained by evaporation at 120 ºC for 60 min.

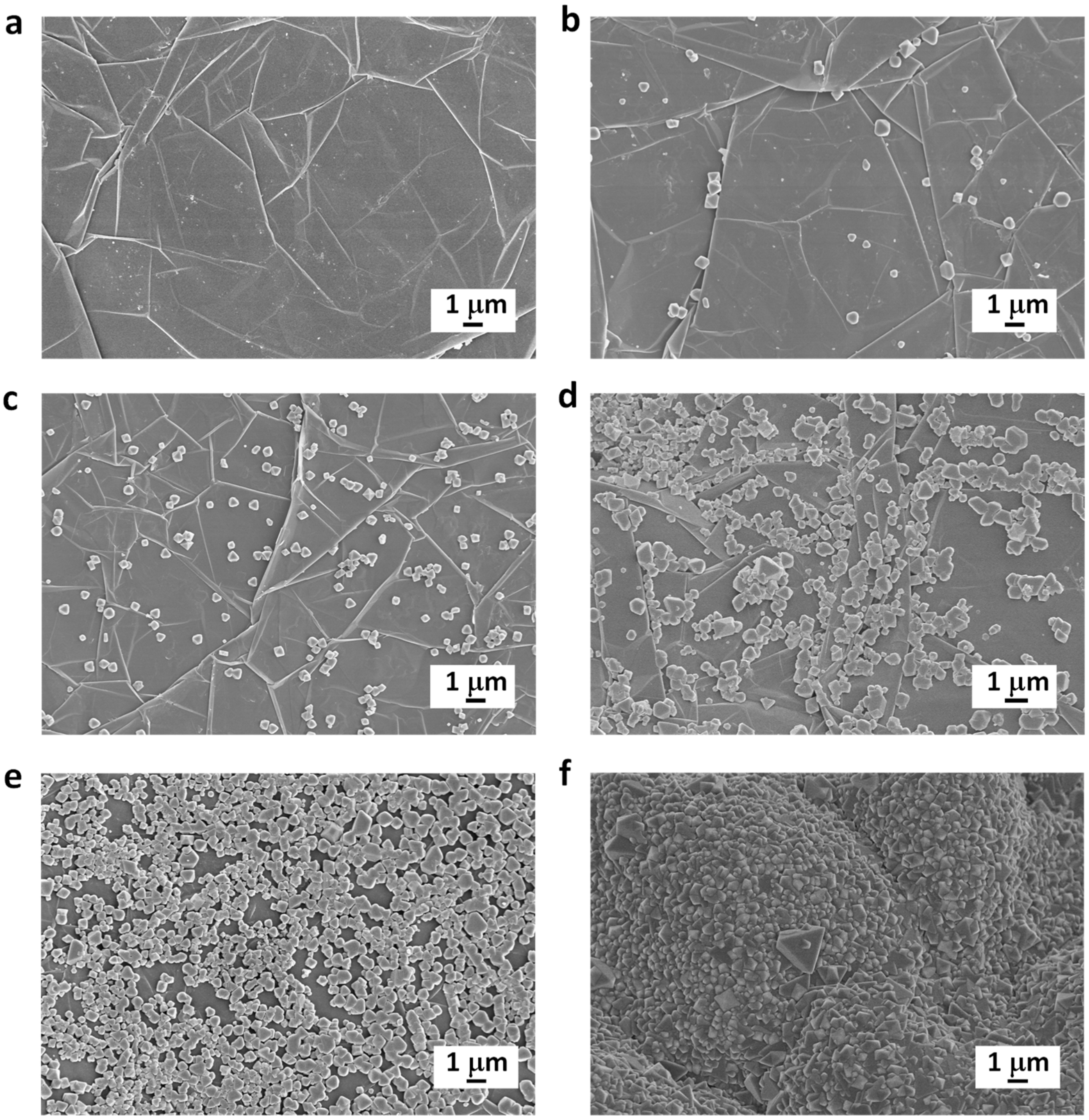

Supplementary Fig. 22 SEM images of HKUST-1/GF obtained with a) 1, b) 2, c) 3, d) 4, e) 5, and f) 6 pulses.

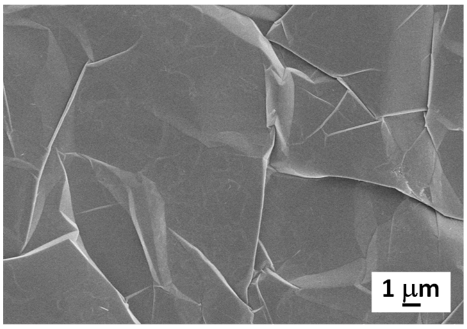

Supplementary Fig. 23 SEM image of the sample obtained by the solvothermal method within ultralow precursor concentrations of 0.85 mM $Cu(NO_3)_2$ and 0.56 mM H_3BTC.

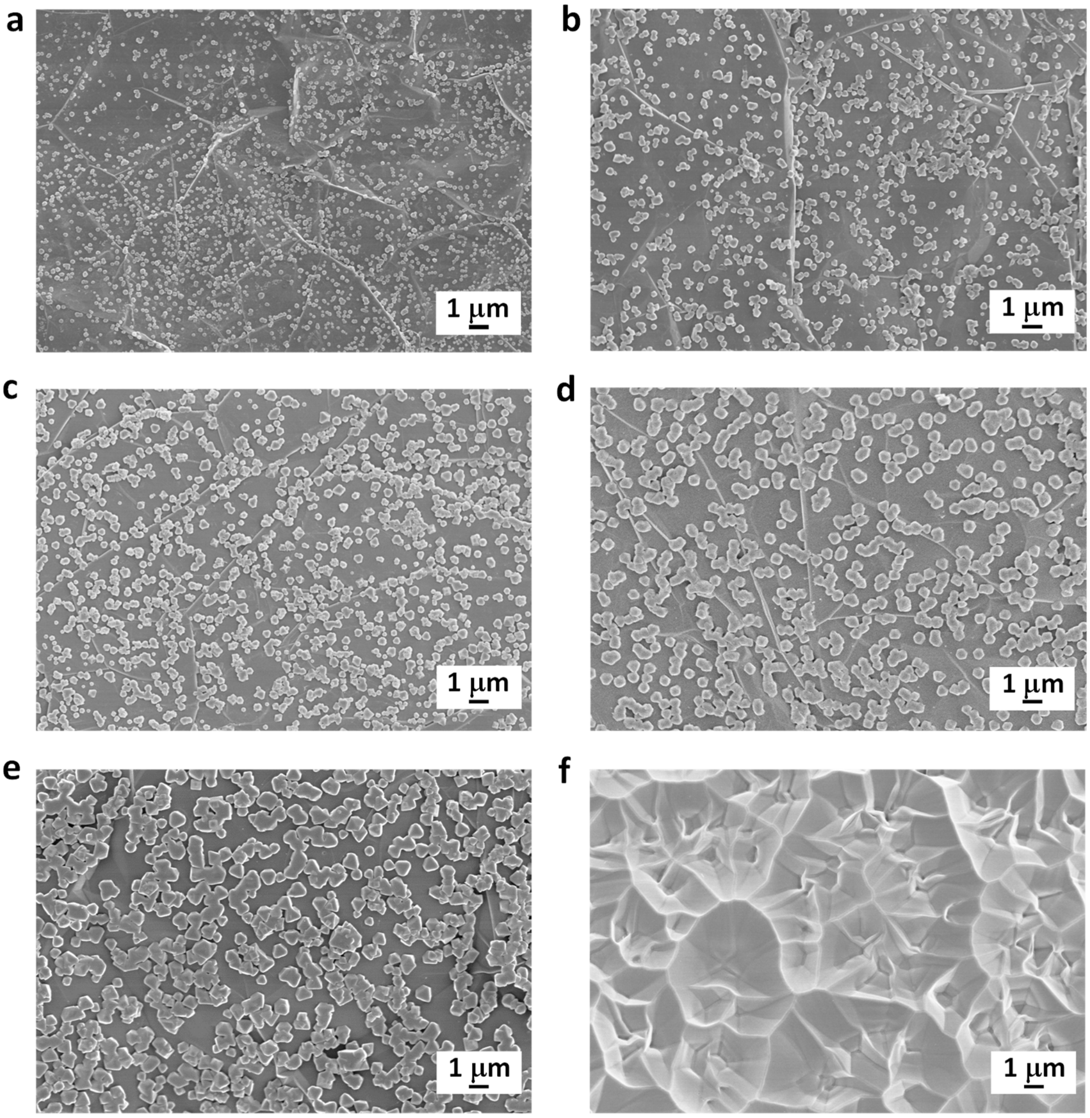

Supplementary Fig. 24 SEM images of HKUST-1/GF obtained within different concentrations of the $Cu(NO_3)_2$ of a) 0.85 mM, b) 1.7 mM, c) 8.5 mM, d) 17 mM, e) 42.5 mM, and f) 170 mM (all with the same 3:2 molar ratio relative to H_3BTC). The average sizes of the HKUST-1 are 207 ± 52, 317 ± 78, 397 ± 86, 552 ± 85, 645 ± 117, and 1208 ± 271 nm, respectively.

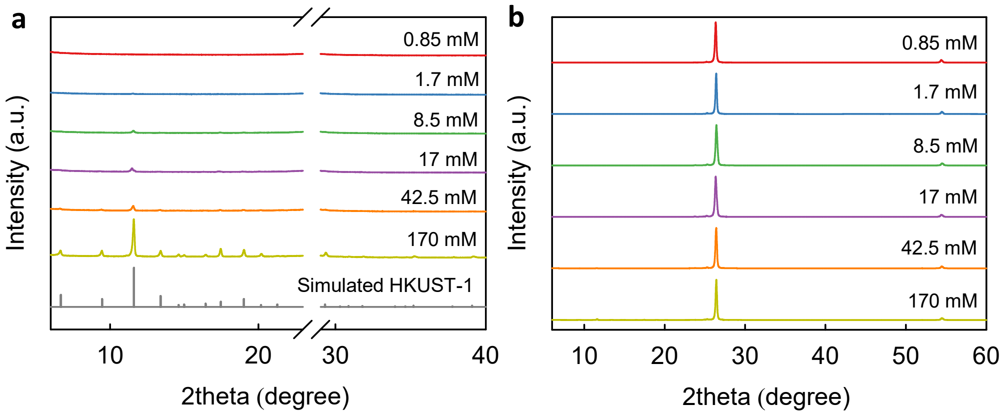

Supplementary Fig. 25 XRD patterns of simulated HKUST-1 and HKUST-1/GF prepared within different initial concentrations of the precursors in the 2θ range of a) 5-40° and b) 5-60°. The sample was distinguished and marked by the concentration of $Cu(NO_3)_2$.

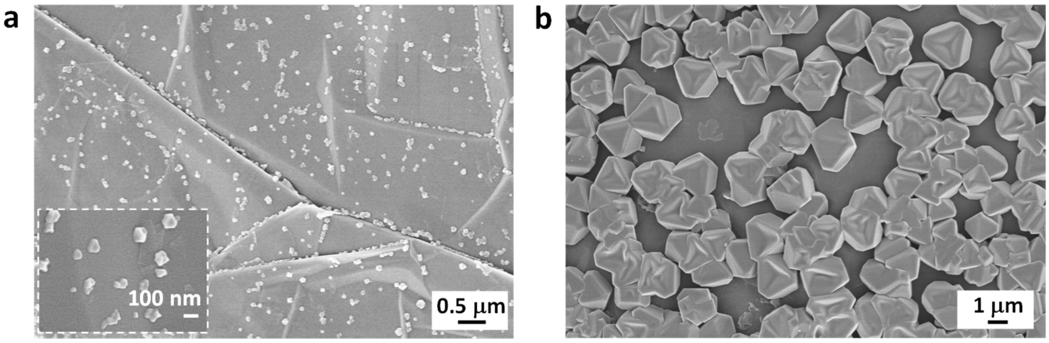

Supplementary Fig. 26 SEM images of a) HKUST-1 nanoparticles obtained within the current of 5.25 A and 0.85 mM $Cu(NO_3)_2$, and b) HKUST-1 microparticles obtained within the current of 1.85 A and 170 mM $Cu(NO_3)_2$ (all with the same 3:2 molar ratio relative to H_3BTC).

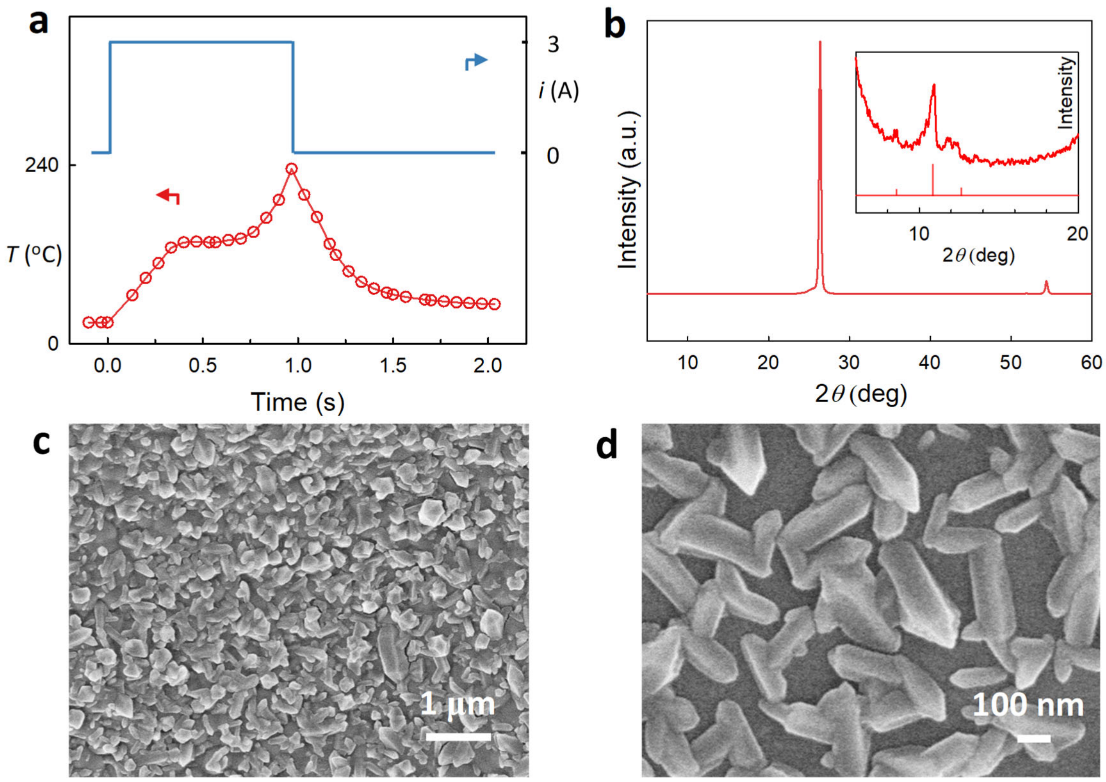

Supplementary Fig. 27 WIJH synthesis and characterizations of MIL-88A(Fe) on the GF. a) Electrified procedure (blue line) and temperature evolution (red line) of the WIJH system. b) XRD pattern of MIL-88A(Fe)/GF. c) and d) SEM images at different magnification scales.

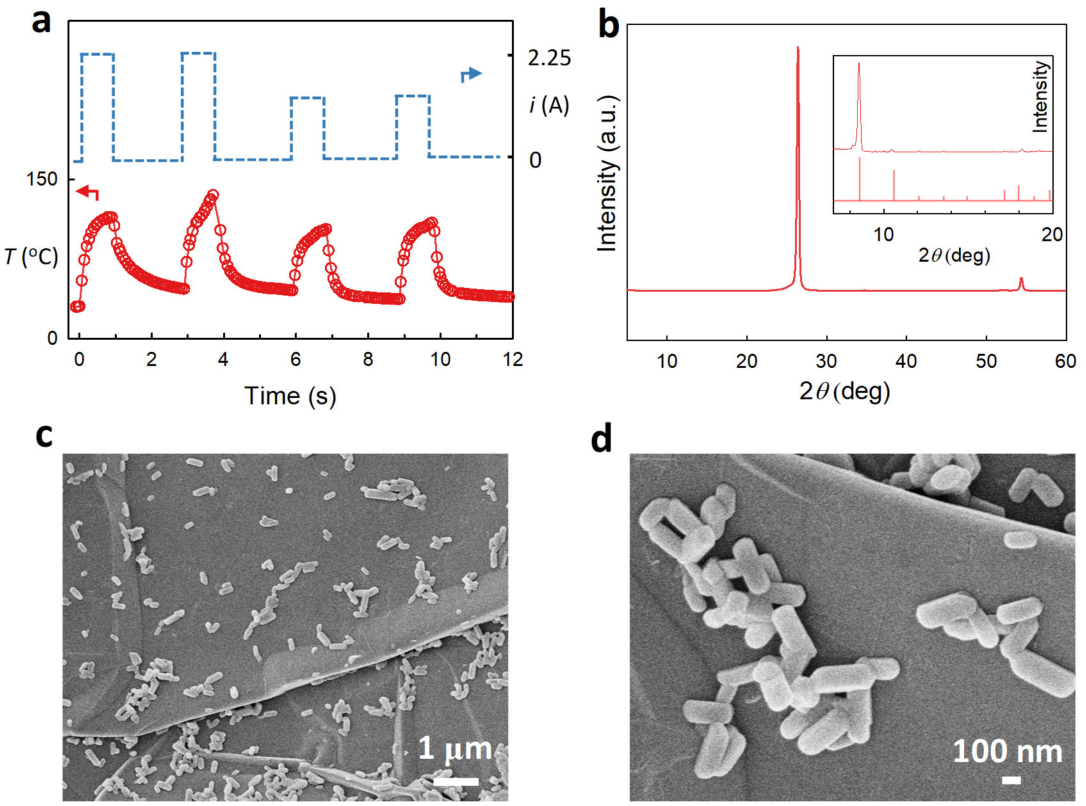

Supplementary Fig. 28 WIJH synthesis and characterizations of Tb-BTC on the GF. a) Electrified procedure (blue line) and temperature evolution (red line) of the WIJH system. b) XRD pattern of Tb-BTC/GF. c) and d) SEM images at different magnification scales.

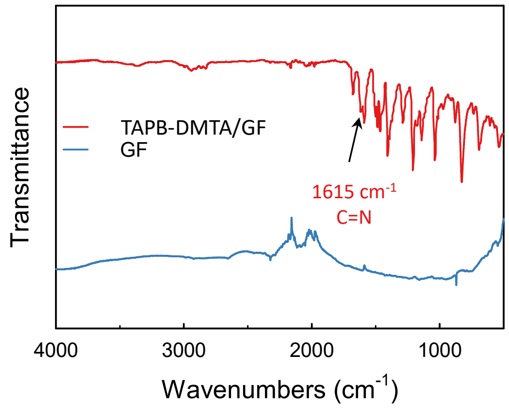

Supplementary Fig. 29 FT-IR spectra of TAPB-DMTA/GF and GF. The appearance of the peak around 1615 cm^{-1} that ascribed to C=N bond confirmed the formation of TAPB-DMTA.

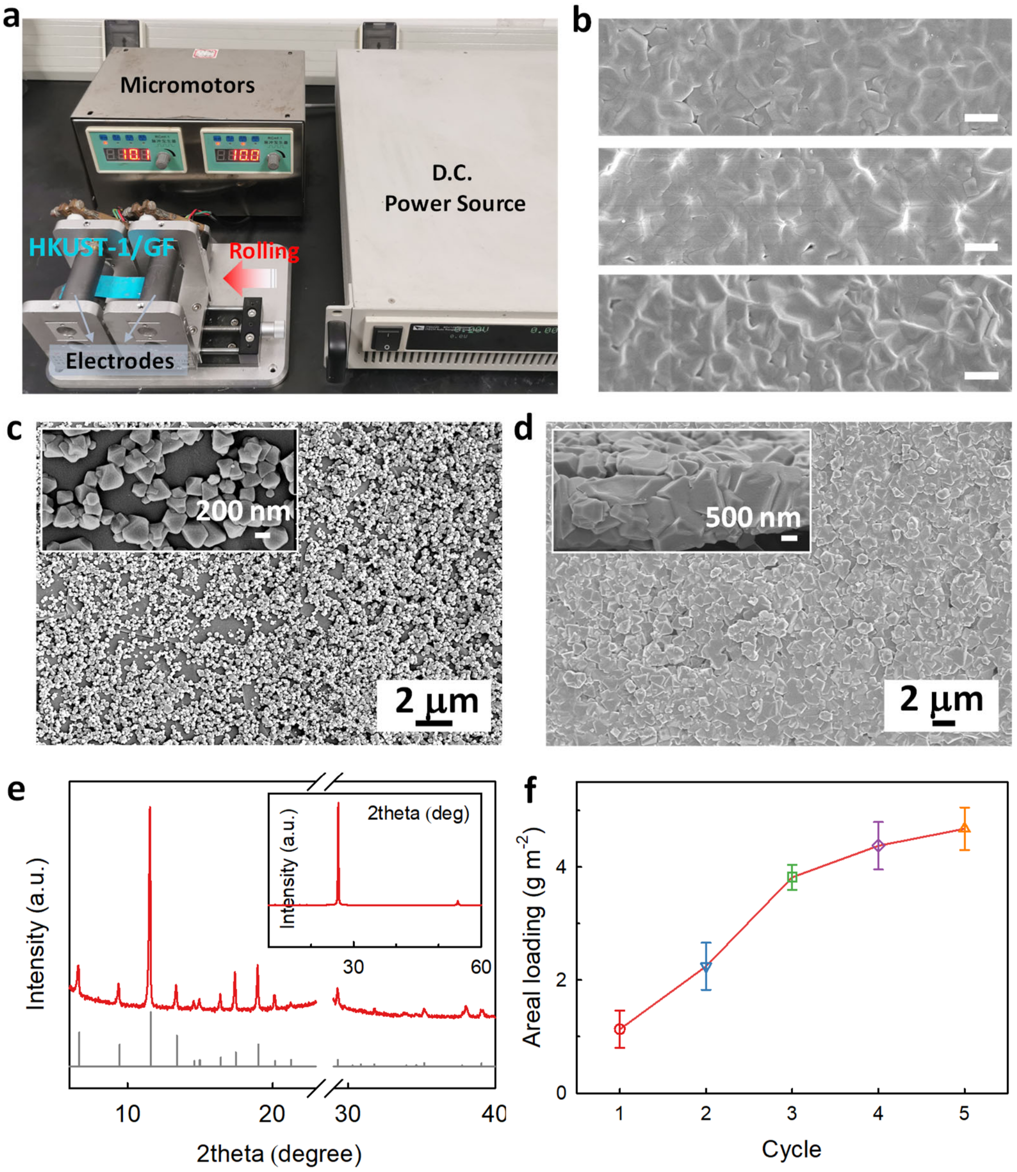

Supplementary Fig. 30 WIJH-based continuous fabrication. a) Photograph of the roll-to-roll Joule-heating setup. b) SEM images of the samples from different sites along the length direction of the upscaled fusing HKUST-1/GF (the distance of each collecting site was about 2 cm), scale bar: 1 µm. SEM images of c) the dispersed HKUST-1 particles and d) multi-layer HKUST-1 coatings on the GF. e) XRD patterns of simulated HKUST-1 (gray) and the fusing HKUST-1/GF (red) within different 2θ ranges. f) Areal loading of HKUST-1 on the GF in the layer-by-layer fashion. Error bars represent the standard deviations of measurement from three samples.

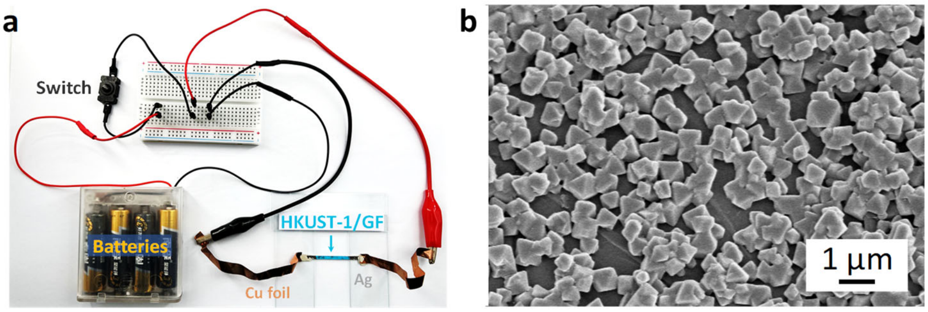

Supplementary Fig. 31 a) Photograph of the portable synthesis setup supplied by the batteries, and b) SEM image of the as-prepared HKUST-1/GF.

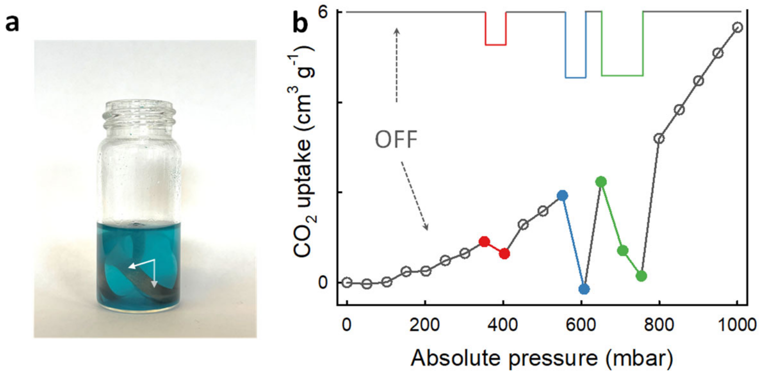

Supplementary Fig. 32 a) Photograph of the HKUST-1/GF synthesized by the solvothermal method. The white arrows indicate the few and nonuniform loading of HKUST-1 on the GF. b) CO_2 adsorption isotherm of HKUST-1/GF prepared by solvothermal synthesis under programmed IJH processes.

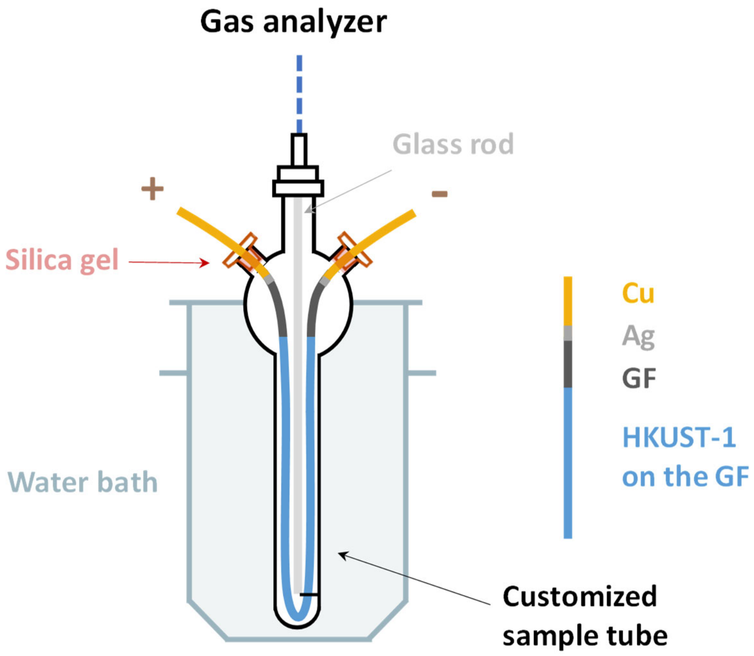

Supplementary Fig. 33 Schematic diagram of the custom-designed IJH setup for the CO_2 adsorption/desorption analysis.

Supplementary Discussion 6

The temperature profile of the WIJH system ranges from 25 to 285 °C (Fig. 2a). This was made up of the temperature of the GF and the solution layer. The temperature values of the GF and the solution change from 25 to 288 °C (Fig. 1c) and ~25 to 153 °C (Fig. 1d), respectively. As the maximum temperature of the solution part is less than the thermal decomposition temperatures of $Cu(NO_3)_2$ (around 200 °C to form copper oxide) and H_3BTC (about 340 °C), precursors will not be decomposed during the WIJH synthesis (Supplementary Fig. 34). To avoid the thermal destruction to the substrate and the product, the temperature of the WIJH system should be less than their thermal decomposition temperatures (~600 °C for the GF and ~300 °C for HKUST-1).

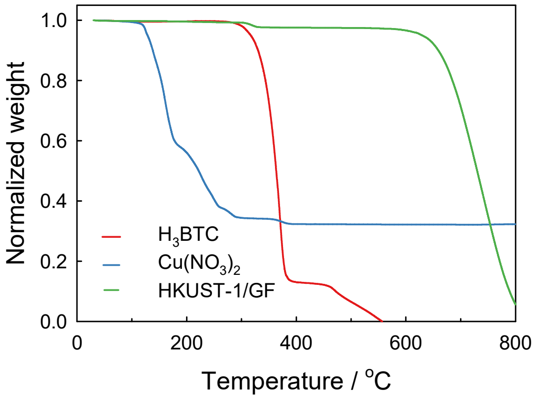

Supplementary Fig. 34 TGA curves of the precursors for HKUST-1 ($Cu(NO_3)_2$ and H_3BTC) and the product of HKUST-1/GF in air.

2. Supplementary Tables

Supplementary Table 1 Analysis results of the N_2 adsorption/desorption isotherms

Materials	Type	BET surface area (m^2 g^{-1})	Average pore size (nm)
HKUST-1/GF	IV	37.556	2.769
GF	IV	14.154	3.969

The average pore size was obtained based on the DFT model.

BET surface area (S_{BET}) of HKUST-1 = ($S_{BET,\ HKUST\text{-}1/GF}$ - $S_{BET,\ GF}$) / L_m.

Supplementary Table 2 Comparison of WIJH strategy used in this work for the synthesis of MOF films with other synthesis methods reported in the literature

Strategies	MOFs	Heating conditions		Synthesis efficiency for a batch			Refs.
		T (°C)	^{a}t (s)	Particle size (nm)	Energy cost (kWh)[a]	Production mass (mg)[b]	
WIJH	HKUST-1	25-285,	0.95<br>0.25	~850<br>~490	2.38×10^{-6}<br>1.91×10^{-6}	1.58-6.54	This work
Solvothermal synthesis	HKUST-1	110	43200	~20000	14.4-42	/	3
Solvothermal synthesis	HKUST-1	120	72000	/	2.63-3.63	/	4
Solvothermal synthesis	UiO-66-NH_2	85	86400	50-100	28.8-84	3.97-5.95	5
Solvothermal synthesis	UiO-66	120	86400	150-500	2.86-4.23	/	6
Solvothermal synthesis	HKUST-1	120	201600	~2350	7.45-10.92	/	7
Microwave	ZIF-8	150	720	~800	1-1.8		
Microwave	MOF-5	120	30	~6500	4.16×10^{-3}	/	8
Microwave	UiO-66	120	1800	~300	0.0175-0.1	/	9
Microwave and evaporation	HKUST-1	90	1860	~170	0.6-1.75	/	10
Oven heating	HKUST-1	120	1800	~800	0.6-1.75	155-930	11
Spray-assisted method	HKUST-1	130	60	~1000	1.4×10^{-4}	/	12
Spray coating	ZIF-67	150	7200	44-5100	4-6	/	13
Vapor-assisted conversion	UiO-66	100	10800	~525	3.6-10.5	/	14
Thermal deposition	ZIF-8	200	900	~1000	0.3-0.88	/	15
Electrical induction heating	HKUST-1	141.3	28800	~2450	0.168	/	16
Hot-pressing	ZIF-8	200	600	~100	0.05-0.17	1.2-3.9	17

Note that the experimental conditions and data to obtain the final product (a MOF coverage rate close to 100%) were recorded.

[a]Energy cost was estimated according to the experimental conditions in the reference and the investigation of the equipment parameters.

[b]Mass was estimated according to the mass loading of MOFs in the final product and the mass of the substrate.

Supplementary Table 3 Statistic results of the synthesis of HKUST-1 on the GF via different heating strategies of 2) solvothermal method, 3) bulk Joule heating, and 4) WIJH

Conditions	Surface coverage ratio (%)	Coverage rate (% s^{-1})	Particle size range (nm)	HKUST-1 crystallization rate (nm s^{-1})
2), 60 min	~6	0.0017	20000-50	5.5-0.01
3), 2 min	25.8	0.22	2600-285	21.7-2.4
4), 0.95 s	100	100	1019-689	1072.6-725.3
4), 0.25 s	100	100	591-391	2364-1564

3. Supplementary References

1 Thanh, N. T., Maclean, N. & Mahiddine, S. Mechanisms of nucleation and growth of nanoparticles in solution. *Chem. Rev.* **114**, 7610-7630 (2014).

2 Bai, S. et al. Laser-assisted reduction of highly conductive circuits based on copper nitrate for flexible printed sensors. *Nano-Micro Lett.* **9**, 42 (2017).

3 Sun, Y. et al. Oriented nano-microstructure-assisted controllable fabrication of metal-organic framework membranes on nickel foam. *Adv. Mater.* **28**, 2374-2381 (2016).

4 Lemaire, P. C. et al. Copper benzenetricarboxylate metal-organic framework nucleation mechanisms on metal oxide powders and thin films formed by atomic layer deposition. *ACS Appl. Mater. Interfaces* **8**, 9514-9522 (2016).

5 Yao, A., Jiao, X., Chen, D. & Li, C. Bio-inspired polydopamine-mediated Zr-MOF fabrics for solar photothermal-driven instantaneous detoxification of chemical warfare agent simulants. *ACS Appl. Mater. Interfaces* **12**, 18437-18445 (2020).

6 Ghalei, B. et al. Rational tuning of zirconium metal-organic framework membranes for hydrogen purification. *Angew. Chem. Int. Ed.* **58**, 19034-19040 (2019).

7 Liu, C. et al. General deposition of metal-organic frameworks on highly adaptive organic-inorganic hybrid electrospun fibrous substrates. *ACS Appl. Mater. Interfaces* **8**, 2552-2561 (2016).

8 Yoo, Y. & Jeong, H. K. Rapid fabrication of metal organic framework thin films using microwave-induced thermal deposition. *Chem. Commun.* 2441-2443 (2008).

9 Appelhans, L. N. et al. Facile microwave synthesis of zirconium metal-organic framework thin films on gold and silicon and application to sensor functionalization. *Micropor. Mesopor. Mat.* **323**, 111133 (2021).

10 Ameloot, R. et al. Direct patterning of oriented metal-organic framework crystals via control over crystallization kinetics in clear precursor solutions. *Adv. Mater.* **22**, 2685-2688 (2010).

11 Gao, G. K. et al. Rapid production of metal-organic frameworks based separators in industrial-level efficiency. *Adv. Sci.* **7**, 2002190 (2020).

12 Kubo, M., Sugahara, T. & Shimada, M. Facile fabrication of HKUST-1 thin films and free-standing MWCNT/HKUST-1 film using a spray-assisted method. *Micropor. Mesopor. Mat.* **312**, 110771 (2021).

13 Chen, Z. et al. Large-area crystalline zeolitic imidazolate framework thin films. *Angew. Chem. Int. Ed.* **60**, 14124-14130 (2021).

14 Virmani, E. et al. On-surface synthesis of highly oriented thin metal-organic framework films through vapor-assisted conversion. *J. Am. Chem. Soc.* **140**, 4812-4819 (2018).

15 Maina, J. W. et al. The growth of high density network of MOF nano-crystals across macroporous metal substrates – Solvothermal synthesis versus rapid thermal deposition. *Appl. Surf. Sci.* **427**, 401-408 (2018).

16 Tao, Y., Huang, G., Li, Q., Wu, Q. & Li, H. Localized electrical induction heating for highly efficient synthesis and regeneration of metal-organic frameworks. *ACS Appl. Mater. Interfaces* **12**, 4097-4104 (2020).

17 Chen, Y. et al. A Solvent-free hot-pressing method for preparing metal-organic-framework coatings. *Angew. Chem. Int. Ed.* **55**, 3419-3423 (2016).
